# Chatbots versus retina specialists in answering real-world retina questions

**DOI:** 10.1186/s40942-025-00737-7

**Published:** 2025-10-31

**Authors:** Mario Cesar Bulla, Caio Augusto Scocco, Viviane Souto Spadoni, Gustavo Matias Hüning, Alexandre Grandinetti, Andre Moraes Freitas, Alexandre Antonio Marques Rosa, Pedro Carlos Carricondo

**Affiliations:** 1 Clinica Bulla, Rua São Joaquim 611, Sala 1203, Centro, Sao Leopoldo, RS CEP 930190 Brazil; 2 Institudo Scocco, Porto Alegre, RS Brazil; 3 Clinica Visao, Porto Alegre, RS Brazil; 4 Instituto Peregrino, Santa Maria, RS Brazil; 5 Hospital de Olhos do Paraná, Curitiba, PR Brazil; 6 Santa Casa de Porto Alegre, Porto Alegre, RS Brazil; 7 Universidade Federal do Pará, Belém, PA Brazil; 8 Universidade de São Paulo, São Paulo, SP Brazil

**Keywords:** Artificial intelligence, Ophthalmology, Retina, Generative artificial intelligence, Chat-GPT, Readability

## Abstract

**Background:**

Chatbots powered by large language models have shown promising results when addressing medical queries, thus transforming access to medical information. Although these chatbots produce detailed and accurate responses, it is unclear how they perform when handling real, unedited patient questions, particularly in non-English languages. This study aimed to assess the readability, accuracy, and comprehensiveness of responses to retinal disease queries provided by four chatbots (ChatGPT 4.0, ConsensusGPT, Gemini, and Claude 3) compared to responses from retina specialists.

**Methods:**

In this cross-sectional, comparative, and blinded study, twenty unedited questions about retinal diseases were randomly selected from a popular online video channel in Portuguese. The questions were submitted to the four selected chatbots and retina specialists with fellowship training. Two independent retinal experts evaluated the responses using standardized Likert scales for accuracy and completeness. Readability was assessed using the Flesch Reading Ease Score and the Flesch-Kincaid Grade Level tests. Additional metrics, including word count and response generation time, were analyzed. Data were compared among groups using non-parametric statistical tests, including the Kruskal-Wallis test with Dunn’s pairwise comparisons and chi-squared tests, with a two-sided *p*-value threshold of 0.05 for statistical significance.

**Results:**

Retinal specialists and the Gemini chatbot produced responses with higher readability, indicating lower educational levels were needed for comprehension. In contrast, ChatGPT 4.0, ConsensusGPT, and Claude 3 delivered more detailed and accurate answers but required a higher reading level. ChatGPT 4.0 and ConsensusGPT achieved superior quality and comprehensiveness ratings compared to human experts and the other chatbots. Additionally, all AI systems generated responses significantly faster than the human specialists. Evaluators could correctly distinguish between human-generated and AI-generated responses in most cases.

**Conclusions:**

Artificial intelligence chatbots demonstrate considerable promise for rapidly disseminating accurate medical information directly to the end-user, not only in English. However, optimizing the simplicity of their language is essential to ensure that detailed responses remain accessible to a broad audience. Future research should aim to replicate our findings with larger datasets of questions with the goal of refining these systems to balance comprehensive content with user-friendly language.

**Supplementary Information:**

The online version contains supplementary material available at 10.1186/s40942-025-00737-7.

## Background

In recent years, large language models (LLMs) have revolutionized how we search for information across various fields, with significant implications in critical areas such as medicine. Among these technologies, natural language chatbots such as ChatGPT have gained immense popularity and have been extensively tested for their reliability in answering medical queries, often producing results that surpass those of medical experts in terms of response quality [1–3]. For instance, a recent study by Huang et al. [4] showed that ChatGPT 4.0 outperformed ophthalmologists when answering questions about glaucoma, while Tailor et al. [5] demonstrated comparable performance when the subject of the questions was retinal diseases.

Other researchers have demonstrated the utility of ChatGPT 4.0 for crafting responses to patient inquiries while highlighting the potential for generating inappropriate and even dangerous responses [5–7]. In addition, several studies have observed that the generation of incorrect content is often presented with the same confidence as accurate information, including the fabrication of non-existent references [4, 8]. Such erroneous outputs pose a significant challenge, as health information accessed online is crucial for doctors and patients seeking to understand their health conditions. Moreover, the fact that people are increasingly turning to the Internet for health information [9] underscores the urgent need to address the issue of erroneous AI-generated responses to medical queries.

Another gap in the medical application of LLM-driven chatbots is that only a few studies have taken into account the layperson’s perspective and communication style, including possible spelling mistakes or misunderstandings about symptoms, which can complicate the acquisition of accurate information, whether from pre-existing online content or through interactions with a chatbot interface. Instead, most studies assessing the reliability of natural language models have used questions posed by medical professionals or patient questions selected and edited by doctors to ensure clarity and relevance [1, 10]. We could only identify two studies evaluating the capacity of LLMs to answer real, unedited questions from the end-user [11, 12]. For instance, Bernstein et al. [12] compared experts versus ChatGPT-3 in answering ophthalmological questions posted by patients in an online medical forum and reported similar results between groups.

Similarly, there is considerable concern about the level of education or reading level required to correctly interpret the answers generated by ChatGPT 4.0 for various medical conditions [13, 14], including retinal diseases [3]. Analysis using readability indexes such as the Flesch-Kincaid Grade Level (FKGL) and Flesch Reading Ease Score (FRES) have shown that the required education level to read and comprehend AI-generated responses to medical queries is well above the recommended level for such situations [7, 10]. One study demonstrated that Bard LLM generated medical texts that are easier to read than those produced by ChatGPT 3.5 and ChatGPT 4.0, as well as institutional material [15].

Furthermore, LLM-driven chat systems have demonstrated proficiency across multiple languages, successfully passing medical examinations and showcasing their potential as decision-support tools for healthcare professionals by instantly processing vast databases [16]. However, we could not find any published articles analyzing the readability of chatbots in Portuguese, even though these readability tests have already been validated for use in Portuguese [17].

Therefore, this study aims to evaluate responses to real, unedited questions posted online in Portuguese about retinal diseases in terms of their readability, accuracy, comprehensiveness, and potential for publication without human interference, comparing five groups: retinal specialists, ChatGPT 4.0 (OpenAI), ConsensusGPT (a search engine, according to its authors, uses ChatGPT 4.0 and a database of 200 million scientific articles), Gemini (Alphabet Inc), and Claude 3 (Anthropic).

## Methods

### Study design

This cross-sectional, comparative, blinded study analyzed the accuracy and completeness of the responses to 20 publicly posted questions about retinal diseases (Supplementary Table 1). The questions were obtained from the popular YouTube Brazilian channel “Retina e Vítreo,” along with the responses provided by the physician managing the channel. They were selected from among the most recent questions available on the channel that addressed topics relevant to the field of retina. These questions, in their original form, in Portuguese, were submitted to the chatbots ChatGPT 4.0 and ConsensusGPT (both paid), Gemini and Claude 3 (free), and to three other ophthalmologists with fellowship training in retina. The collection of responses from the chatbots was conducted entirely by the primary researcher between 15 March and 25 April 2024.

### Chatbot queries

The following prompt was inserted before each question: “*I am an ophthalmologist*,* and you will help me answer questions from my followers. In your response*,* be relatively succinct and ethical; we cannot recommend procedures without an examination but can clarify doubts. You should not say that you are not a doctor in the response because I will use it as if I had answered. I will paste the question here*:” Despite the prompt specifying the requirement for concise answers, the responses generated by Gemini were much longer than the other groups. Therefore, this chatbot was configured in its settings to provide short responses. A new chat was used for each question to avoid any information crossover.

### Experts’ response

After reading and signing the informed-consent form, specialist physicians were asked to read the questions and respond without consulting LLMs. They received the following instructions: *I am an ophthalmologist*,* and you will help me answer 20 questions from my followers. In your responses*,* be concise and ethical; do not recommend procedures without a proper examination*,* but clarify any doubts.* Physicians were also instructed to record, in seconds, the time from finishing reading each question to completing their written answer. It was not necessary to answer the questions sequentially.

### Response evaluation

The quality of the responses was evaluated in a blinded and randomized manner by two other ophthalmologists, also with fellowship training in retina and extensive experience in the field, using standardized Likert scales, one for accuracy and another for completeness of the response, as well as additional questions regarding whether the response appeared to have been generated by a human or artificial intelligence, and whether such a response could be publicly posted without causing harm to the patient or ethical issues. The evaluations of both ophthalmologists were considered together for all statistical analysis. The reliability between raters was assessed through correlation analysis of the scores assigned by the two independent evaluators.

The accuracy scale was a 10-point Likert scale (1 unacceptable, 2 slightly better than 1, 3 poor response with serious errors, 4 slightly better than 3, 5 some errors, 6 slightly better than 5, 7 good, 8 slightly better than 7, 9 excellent, and 10 better than 9). The completeness scale was a 3-point Likert scale with 1 indicating incomplete (i.e., addresses some aspects of the question but significant parts are missing or incomplete), 2 indicating adequate (i.e., addresses all aspects of the question and provides the minimum amount of information necessary to be complete, and 3 indicating comprehensive (i.e., addresses all aspects of the question and provides additional information or context beyond what is expected) response. Additionally, we analyzed whether question type influenced quality scores by categorizing the 20 assessment questions as judgment-based (*n* = 10) or factual (*n* = 10) and comparing quality scores between these categories across all evaluation groups.

FKGL and FRES were used to assess the readability of the responses. These tests estimate the average educational level required to understand a text and its ease of reading; such indices have already been validated for use in Portuguese [17], and their methodology is explained elsewhere [18]. Calculations were performed using an online calculator (https://goodcalculators.com/flesch-kincaid-calculator/), where it is also possible to obtain the number of words per response. The interpretation scheme used in the FRES scale is described in Table 1. In addition, we evaluated response length (word count) and response time (self-recorded by physicians and researcher-measured for chatbots), as well as potential correlations between word count and both quality and completeness scores.

**Table 1:** Interpretation scheme of Flesch Reading Ease Score

Flesch reading ease	Description of style	Educational attainment level (US)
0–30	Very difficult	Postgraduate
30–50	Difficult	Undergraduate
50–60	Fairly difficult	Grade 10–12
60–70	Standard	Grade 8–9
70–80	Fairly easy	Grade 7
80–90	Easy	Grade 6
90–100	Very easy	Grade 5

### Sample size calculation

For the readability analysis, a sample size of 100 per group was determined to achieve 80% power for detecting a 20% difference between groups; therefore, we collected an additional 80 questions with their respective answers, which were submitted to the chatbots, totaling 100 responses for each chatbot, as well as 160 human responses (60 from specialists, 100 from the channel itself).

### Statistical analysis

Data were presented as median and interquartile ranges (IQR). Statistical analyses included: Kruskal-Wallis test for inter-group comparisons with Dunn’s post hoc test for pairwise comparisons, Kendall’s tau for inter-rater reliability, Mann-Whitney U test for comparing quality scores between factual and judgment-based questions, and Spearman correlation for examining relationships between word count and Likert scores. The structured questions (possibility of publication and origin of the responses) were compared using the Chi-squared test. Whether the answer came from experts or artificial intelligence was evaluated using a contingency table. A two-sided *p*-value < 0.05 was considered statistically significant. Statistical analysis was conducted using SigmaPlot^®^ Version 15.0 (Grafiti LLC) and Python (version 3.11.8; Python Software Foundation) with the SciPy statistical package (version 1.14.1) executed in the Spyder environment (version 5.4.1).

## Results

### Flesch readability ease score

**Fig. 1:**
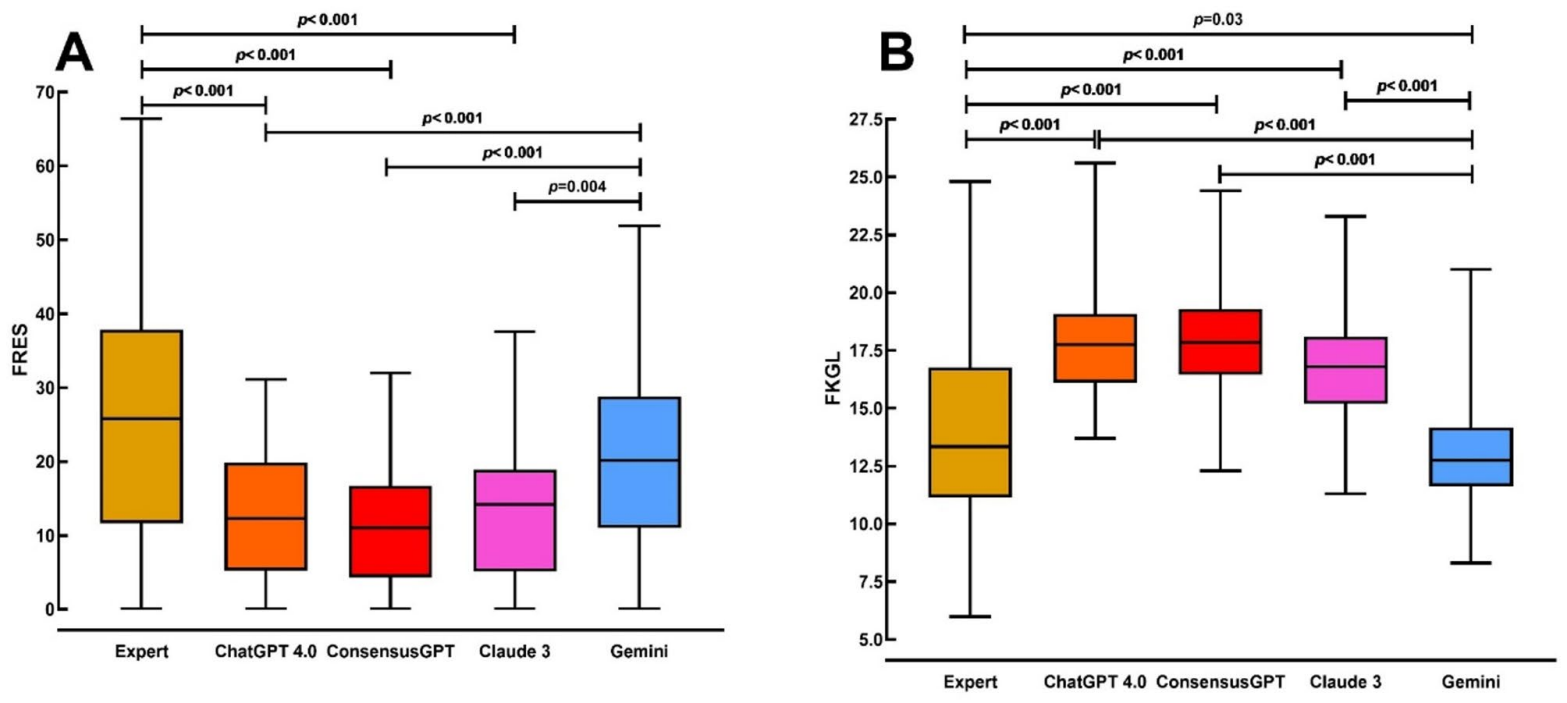
Pairwise comparisons of the median (**A**) Flesch reading ease score and (**B**) Flesch-Kincaid grade level of responses generated by human experts, ChatGPT 4.0, ConsensusGPT, Claude 3, and Gemini using Dunn’s method

The group of answers provided by experts had a median FRES of 25.8 (IQR: 11.6–37.9), while responses from Gemini had a median score of 20.1 (IQR: 11.0–28.1) with no inter-group differences (*p* = 1.0) (Fig. 1A). However, both experts and Gemini had significantly higher FRES values, indicating easier readability compared to ChatGPT 4.0 [12.3 (IQR: 5.2–19.9)], ConsensusGPT [11.0 (IQR: 4.3–16.7)], and Claude 3 [14.2 (IQR: 5.1–18.9)] (*p* < 0.001 for all comparisons except Gemini vs. Claude 3 where *p* = 0.004) (Fig. 1A). There was no statistical difference between the median FRES of ChatGPT 4.0, ConsensusGPT, and Claude 3 (*p* > 0.05).

### Flesch-Kincaid grade level

The experts’ responses had a median FKGL of 13.3 (IQR: 11.1–16.8), while Gemini had a median FKGL of 12.7 (IQR: 11.6–14.2), with a significant difference between these two groups (*p* = 0.03) (Fig. 1B). Both experts and Gemini had significantly lower FKGL values compared to ChatGPT 4.0 [17.7 (IQR: 16.1–19.1)], ConsensusGPT [17.8 (IQR: 16.4–19.3)], and Claude 3 [16.8 (IQR: 15.2–18.1)] (*p* < 0.001 for all) (Fig. 1B). No significant differences were found among ChatGPT 4.0, ConsensusGPT, and Claude 3 (*p* > 0.05 for all).

### Assessment of answer quality

**Fig. 2:**
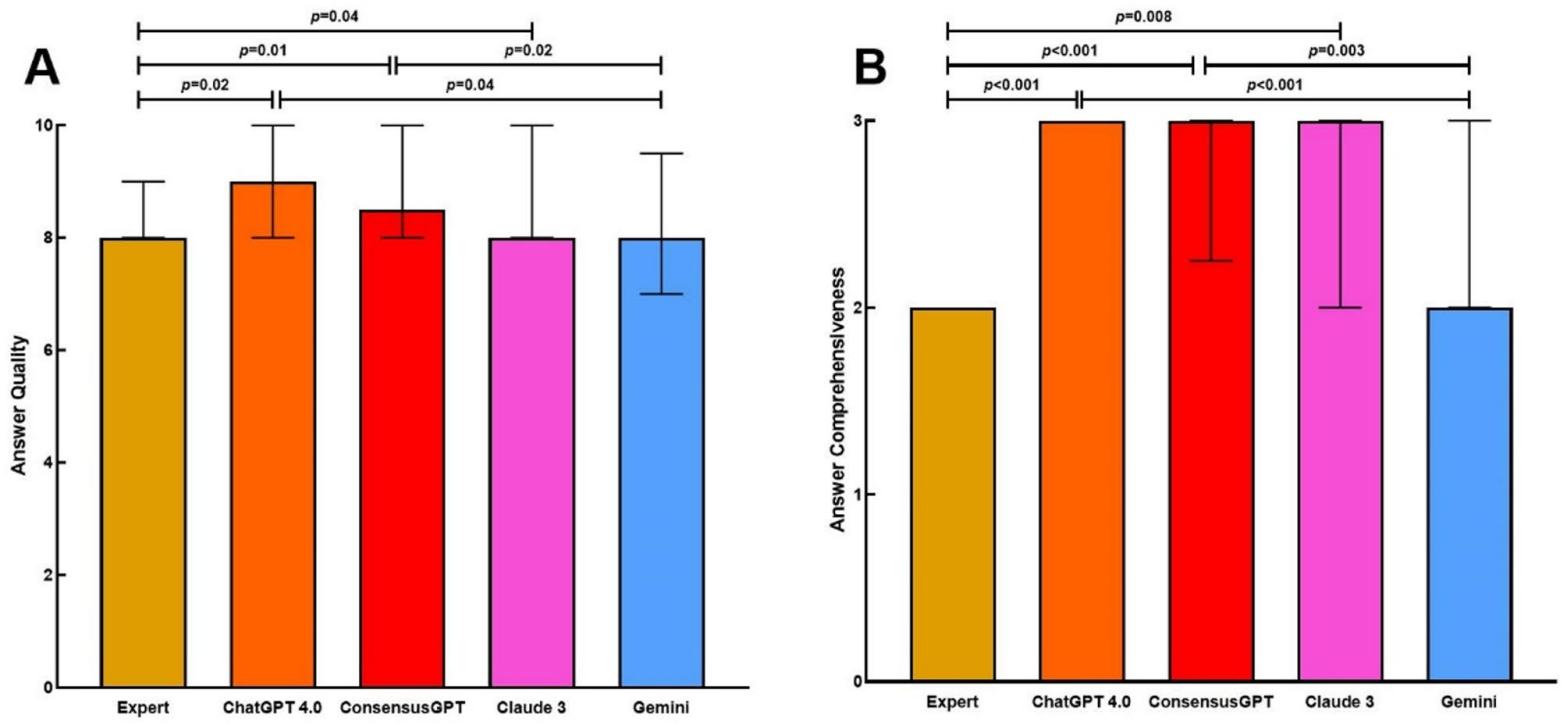
Pairwise comparisons of the median answer (**A**) quality and (**B**) comprehensiveness generated by human experts, ChatGPT 4.0, ConsensusGPT, Claude 3, and Gemini using Dunn’s method. Bars represent medians with whiskers showing interquartile ranges (IQR)

ChatGPT 4.0 achieved a median quality score of 9.0 (IQR: 8.0–10.0), and the ConsensusGPT group obtained 8.5 (IQR: 8.0–10.0) (Fig. 2A). Both ChatGPT 4.0 and ConsensusGPT demonstrated significantly higher quality scores compared to the experts [8.0 (IQR: 8.0–9.0)] and Gemini [8.0 (IQR: 7.0–9.5)]. Pairwise Dunn test revealed the following *p*-values for these comparisons: ChatGPT 4.0 vs. experts (*p* = 0.02), ChatGPT 4.0 vs. Gemini (*p* = 0.04), ConsensusGPT vs. experts (*p* = 0.01), and ConsensusGPT vs. Gemini (*p* = 0.02). Additionally, Claude 3 [8.0 (IQR: 8.0–10.0)] showed significantly higher quality scores than the experts (*p* = 0.04). No significant differences were observed between ConsensusGPT and ChatGPT 4.0, ConsensusGPT and Claude 3, ChatGPT 4.0 and Claude 3, or Claude 3 and Gemini (*p* > 0.05 for all).

Inter-rater reliability analysis using Kendall’s tau demonstrated moderate concordance between evaluators (τ = 0.317, *p* < 0.001).

No significant differences in quality scores were observed between factual and judgment-based questions when considering the entire sample, as well as within each evaluation group (experts, ChatGPT 4.0, Consensus, Gemini, and Claude 3 groups; all *p* > 0.05).

To illustrate cases with low scores that could potentially raise concerns, Table 2 presents the lowest-scoring responses from each group, including experts. Answers with scores 5 and below were included, with the exception of ChatGPT 4.0 and ConsensusGPT, whose lowest scores were 6 and 7, respectively; these responses were therefore included.

**Table 2:** Responses with the lowest quality scores

Author	Question Number	Score	Question	Answer	Type
Expert	1	5	Doctor, I removed the silicone oil a month ago, and my vision is still not good. Is this normal?	It’s not normal but it can happen if some gaseous vitreous substitute was used or in cases where some complication occurred, such as vitreous hemorrhage or retinal detachment. The ideal is always to have close follow-up with the surgeon.	J
Expert	2	1	Doctor, good afternoon. My father was informed that he has bleeding in his eyes. What can be done, Doctor? They told him to do laser treatment, but he says he feels pain when he goes to do this laser and doesn’t continue the treatment. I feel very sad about this because I know that at any moment he could lose his vision. He’s already elderly, 68 years old, and insists on riding a motorcycle. I don’t know what to do.	It would be good to consult so we can see!! Thank you very much for the comment! Don’t forget to share our videos so we can help more people! Hug!	J
Expert	5	4	I have retinitis pigmentosa. Is there any supplement, medication, or procedure to improve or reverse my vision?	Hello! Retinitis pigmentosa is a genetic disease that appears in early adulthood and usually affects both eyes. Previously, vitamins were recommended to help delay the disease progression. Recent studies have shown that vitamins actually worsened the case. Currently, there is research involving retinal chip implants and therapy to improve the quality of life of these patients in the future.	F
Expert	5	5	I have retinitis pigmentosa. Is there any supplement, medication, or procedure to improve or reverse my vision?	Retinitis pigmentosa is a serious disease that can lead to blindness. Currently, there are no treatments that can reverse vision loss caused by the disease itself, except for its complications such as cataracts and epiretinal membranes. Some studies suggest that vitamin A supplementation and a diet rich in cold-water fish may slow the disease.	F
Expert	8	5	Good afternoon, Doctor. I have 10% vision, I removed the silicone oil 8 months ago. Could my vision still improve with more time?	The ideal is to schedule an evaluation appointment with your ophthalmologist to assess the cause of low vision and whether it may have any treatment indication.	J
Expert	12	5	Hello doctor. I have symptoms of detachment. What should I do until I go to the ophthalmologist?	Hello. If the patient has retinal detachment, it is important to avoid sudden head movements and unnecessary movement. The influx of fluid under the retina can be increased in these situations, which will cause an increase in the detached area, increasing the difficulty of possible treatment.	F
Expert	13	4	Hi, how are you? I’m 14 years old and a few weeks ago I pointed a 2000w laser at my right eye out of curiosity, and now I have a very small blurred spot in the center of my right vision. What do I do now?	You should consult with an ophthalmologist to evaluate your retina.	J
Expert	16	5	Hello! I had central retinal vein occlusion on 04/07/23 and macular edema. Since then, I have monthly intravitreal anti-VEGF injections. After the first 3 applications, I had recurrent macular edema. My doctor verified on OCT that I have an epiretinal membrane. Can this membrane interfere with treatment? Is surgery indicated in this case? It should be noted that I have advanced glaucoma in both eyes and amaurosis in the left eye.	Epiretinal membrane may have surgical indication, depending on each case, but the indications depend on evaluation with your retina specialist ophthalmologist.	J
ChatGPT 4.0	4	6	Hello doctor, in August I had a retinal detachment and had oil placed. Now in January it’s scheduled to be removed, but my doctor said it would be good to place gas as soon as the oil is removed. This worries me a bit. Is it normal to remove oil and place gas?	The use of silicone oil or gas (such as sulfur hexafluoride SF6 or perfluoropropane C3F8) in retinal detachment treatment is a common and recognized practice. These materials act as a kind of “spare tire,” keeping the retina in place after the retinopexy procedure, which can be performed with laser or cryotherapy, depending on the case. The replacement of silicone oil with gas may be recommended for several reasons, including the effectiveness of gas in keeping the retina attached during the healing process, in addition to allowing faster absorption compared to oil. This means that in many cases, the gas naturally dissipates from the eye after a period, eliminating the need for a second surgery for its removal. It is important to discuss with your doctor the specific risks and benefits of this procedure in your case, including why gas use is recommended after oil removal, recovery expectations, and any specific precautions you should take during the recovery process. Open communication with your doctor will help clarify your doubts and make the treatment process as smooth as possible.	J
ConsensusGPT	18	7	After applying Avastin, can there be retinal detachment?	After Avastin (Bevacizumab) application, some studies have reported the development or progression of retinal detachment, especially in patients with severe proliferative diabetic retinopathy. In one study, it was identified that 11 eyes out of 211 intravitreal Avastin injections (5.2%) developed or had progression of tractional retinal detachment. All eyes had proliferative diabetic retinopathy refractory to panretinal photocoagulation, and retinal detachment occurred or progressed on average 13 days after injection (Arevalo et al., 2007). This study suggests that tractional retinal detachment may occur or progress after intravitreal bevacizumab administration in patients with severe proliferative diabetic retinopathy. It is important to monitor patients closely after Avastin injection, especially those with severe proliferative diabetic retinopathy, to identify and treat any retinal detachment progression as quickly as possible.	F
Claude 3	11	1	Good afternoon. Can stress cause retinal detachment?	Yes, stress can indirectly contribute to retinal detachment in some cases. Prolonged stress is associated with increased blood pressure and internal ocular pressure (intraocular pressure). When intraocular pressure is very high, it can lead to problems such as retinal detachment, especially in predisposed people or those with other underlying ocular conditions. However, it is important to note that retinal detachment generally involves other risk factors, such as ocular trauma, severe myopia, previous ocular surgeries, degenerative retinal diseases, among others. Stress alone is not a direct cause, but can potentiate risks when associated with these other factors. Therefore, although stress is not the main cause, adequate stress control is recommended through activities such as meditation, physical exercise, and relaxation techniques. This can help maintain ocular pressure at healthy levels and reduce the risk of ocular complications.	F
Gemini	1	5	Doctor, I removed the silicone oil a month ago, and my vision is still not good. Is this normal?	Hello! Vision recovery after silicone oil removal varies greatly, as it depends on several factors, such as: Retinal health before surgery: Attached macula: greater chance of good vision. Detached macula: more limited recovery. Severity of retinal detachment: Mild cases: good chance of normal vision. Severe cases: more challenging recovery. Duration of silicone oil implant: Short (up to 3 months): lower risk of complications. Long (more than 6 months): increased risks. Previous surgeries and general ocular health: Previous surgeries and ocular diseases can affect final vision. Quality of removal surgery and recovery time: Well-performed surgery and adequate recovery optimize results. Systemic conditions: Diseases like diabetes can affect recovery. Observations: Uncertain individual result: Various factors interrelate. Ophthalmologic evaluation: Fundamental to determine prognosis. Post-operative follow-up: Essential to optimize results. Remember: Guide the patient about factors that influence recovery. Be realistic in expectations. Follow the patient closely. By following these guidelines, you will be able to provide accurate and ethical information to your patients.	J
Gemini	7	5	Can Central Serous lead to blindness?	Central Serous Chorioretinopathy (CSC) rarely leads to blindness. In most cases, vision resolves spontaneously. Ophthalmologic consultation is fundamental for individualized evaluation and follow-up. In case of doubts, seek an ophthalmologist.	F
Gemini	19	3	Good morning! What type of gas is used in the eye?	Gases in ophthalmology: Retinal surgeries: Sulfur hexafluoride (SF6): common, repositions detached retina. Octafluoropropane (C3F8): alternative, longer duration of action. Diagnosis: Cyclopentane: fundus examination, temporarily replaces vitreous humor. Observations: Gas choice depends on surgery and patient. Application by ophthalmologist in surgical environment. Possible side effects: blurred vision, changes in ocular pressure and refraction imbalance. Remember: This answer does not replace individualized medical consultation. Consultation with ophthalmologist is fundamental for adequate diagnosis and treatment.	F

Lowest-scoring responses from each group, with question type classified as F (factual) or J (judgment-based). Notable examples include the response to question 11 provided by Claude 3 and the response to question 19 provided by Gemini

### Answer comprehensiveness

In the comprehensiveness assessment (Fig. 2B), ChatGPT 4.0 [3.0 (IQR: 3.0–3.0)], ConsensusGPT [3.0 (IQR: 2.2–3.0)], and Claude 3 [3.0 (IQR: 2.0–3.0)] achieved a similar median score indicating significantly higher comprehensiveness than the experts [2.0 (IQR: 2.0–2.0)]. In addition, ChatGPT 4.0 and ConsensusGPT showed significantly higher comprehensiveness than the Gemini [2.0 (IQR: 2.0–3.0). Pairwise Dunn test revealed the following *p*-values for these comparisons: ChatGPT 4.0 vs. experts (*p* < 0.001), ChatGPT 4.0 vs. Gemini (*p* < 0.001), ConsensusGPT vs. experts (*p* < 0.001), ConsensusGPT vs. Gemini (*p* = 0.003), and Claude 3 vs. experts (*p* = 0.008). No significant differences were found between ChatGPT 4.0 and ConsensusGPT, ChatGPT 4.0 and Claude 3, ConsensusGPT and Claude 3, or between Claude 3 and Gemini (*p* > 0.05 for all).

Inter-rater reliability analysis using Kendall’s tau coefficient showed moderate positive correlation for completeness ratings (τ = 0.337, *p* < 0.001).

### Potential for publication

According to the opinions of the two retina specialists who evaluated the responses, 140 out of 160 (87.5%) evaluations of expert responses could be published without correction, while 20 were deemed inadequate (Table 3). For ChatGPT 4.0, 38 out of 40 responses (95%) were suitable for publication. For ConsensusGPT, in all 40 evaluations (100%), the response could be published without prior intervention. For Claude 3, 38 out of 40 (95%) could be published, while for Gemini, 37 out of 40 (92.5%) could be published. The comparison between the groups using Pearson’s chi-squared demonstrated no statistically significant difference (*p* = 0.08). Pairwise comparisons were performed using Fisher’s exact test to examine differences among the five groups. Ten pairwise tests were conducted, and the Bonferroni correction was applied to control for Type I errors due to multiple comparisons. Although the comparison between expert and ConsensusGPT initially yielded a raw *p*-value of 0.02, the corrected *p*-value increased to 0.16. Ultimately, none of the pairwise comparisons achieved statistical significance.

### Distinction between human responses and artificial intelligence

An analysis was conducted to assess whether readers could accurately distinguish between human responses and those produced by LLMs. A contingency table was constructed by comparing the true origins of the responses with the readers’ guesses (Table 3). The results showed that, out of 160 evaluated human-generated responses, readers correctly identified 154 as human-generated (96.3%) and misclassified six as AI-generated. For AI-generated responses, 112 were accurately recognized, while 48 were mistakenly attributed to human origin. This resulted in an overall accuracy of 83.1%, with a precision of 76.2% for human responses and a sensitivity of 96.2%, suggesting that readers were particularly adept at identifying human-generated content. A chi-squared test for independence yielded a statistic of 150.86 with 1 degree of freedom and a *p*-value < 0.0001, indicating a strong association between the actual sources of the responses and the readers’ classifications and demonstrating that readers distinguished between human and AI-generated responses at a rate significantly better than chance. When comparing pairwise, all human expert versus AI chatbot comparisons showed statistically significant differences (*p* < 0.001, Chi-squared test with Bonferroni correction). Among inter-model comparisons, only ConsensusGPT versus Claude 3 (*p* = 0.0045) and ConsensusGPT versus Gemini (*p* = 0.038) reached significance (Chi-squared test with Bonferroni correction).

**Table 3:** Proportion of responses produced by experts and AI chatbots that could be published without prior intervention and the reader’s ability to distinguish the source of the response

AI Chatbots	Potential for Publication		Distinction between human & AI response	
	Yes	No	Human	AI
Expert	140 (87.5%)	20 (12.5%)	154 (96.3%)	6 (3.8%)
ChatGPT 4.0	38 (95%)	2 (5%)	11 (27.5%)	29 (72.5%)
ConsensusGPT	40 (100%)	0 (0%)	4 (10%)	36 (90%)
Claude 3	38 (95%)	2 (5%)	18 (45%)	22 (55%)
Gemini	37 (92.5%)	3 (7.5%)	15 (37.5%)	25 (62.5%)

All values are presented as n (%)

### Response length

**Fig. 3:**
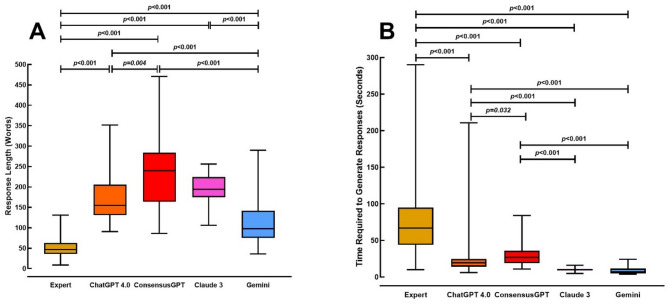
Pairwise comparisons of the median (**A**) response length and (**B**) time required to generate the response by human specialists, ChatGPT 4.0, ConsensusGPT, Claude 3, and Gemini using Dunn’s method

To assess the size of the responses, we measured the number of words in each answer (Fig. 3A). Kruskal-Wallis test revealed a statistically significant difference in response length among the groups (H = 346.1, df = 4, *p* < 0.001). The median word counts were as follows: experts produced responses with a median of 47 words (IQR: 36.2–63.0); ChatGPT 4.0 generated responses with a median of 154.5 words (IQR: 131.0–205.7); ConsensusGPT produced the longest responses with a median of 239.5 words (IQR: 163.7–284.0); Claude 3 yielded a median of 194.5 words (IQR: 175.0–224.5); and Gemini responses had a median of 97.5 words (IQR: 75.0–142.0).

Post-hoc pairwise comparisons using Dunn’s method indicated that ConsensusGPT, ChatGPT 4.0, and Claude 3 produced significantly longer responses than experts (*p* < 0.001) and Gemini (*p* < 0.001). Gemini responses were significantly longer than those from experts (*p* < 0.001). Significant differences were also observed between ConsensusGPT vs. ChatGPT-4 (*p* = 0.004). No significant differences in response length were found between ConsensusGPT vs. Claude 3 or Claude 3 vs. ChatGPT 4.0 (*p* > 0.05 for both).

Spearman correlation analysis revealed a statistically significant positive correlation between word count and Likert quality scores in the overall sample (ρ = 0.3587, *p* < 0.001, 95% CI [0.2593, 0.4506]), indicating a medium effect size. This relationship was primarily driven by the expert group (ρ = 0.3734, *p* < 0.001, 95% CI [0.2316, 0.4996]), while AI systems showed weaker, non-significant correlations (ρ = 0.0396 to 0.1900, all *p* > 0.05). Similarly, analysis revealed a statistically significant large positive correlation between word count and completeness scores in the overall sample (ρ = 0.5352, *p* < 0.001, 95% CI [0.4521, 0.6091]). Group-specific analyses showed that this relationship was driven primarily by the expert group, which demonstrated a significant medium positive correlation (ρ = 0.4006, *p* < 0.001, 95% CI [0.2617, 0.5233]), and the Gemini group, which showed a marginally significant correlation (ρ = 0.3121, *p* = 0.050, 95% CI [0.0007, 0.5684]). Other AI systems showed weaker, non-significant correlations (ρ = -0.0401 to 0.2603, all *p* > 0.05).

### Time required to generate responses

The time taken to generate responses was evaluated by measuring the duration of each answer (in seconds) (Fig. 3B). Kruskal-Wallis test showed a statistically significant difference in response times among the groups (H = 369.8, df = 4, *p* < 0.001). The median response times) for each group was as follows: Experts: 67 s (IQR: 44–95 s), ChatGPT 4.0: 19.5 s (IQR: 14.0–24.7 s), ConsensusGPT: 27 s (IQR: 19–36 s), Claude 3: 10 s (IQR: 9–11 s), Gemini: 7 s (IQR: 5–11.5 s).

Post-hoc pairwise comparisons using Dunn’s method demonstrated that the response times for human experts were significantly longer than those for all AI systems (*p* < 0.001 for comparisons between experts and each chatbot). Among the AI systems, we noted significant differences between ConsensusGPT vs. Gemini (*p* < 0.001), ConsensusGPT vs. Claude 3 (*p* < 0.001), ConsensusGPT vs. ChatGPT-4 (*p* = 0.032), ChatGPT-4 vs. Gemini (*p* < 0.001), and ChatGPT-4 vs. Claude 3 (*p* < 0.001). There was no significant difference between the response times of Claude 3 and Gemini (*p* = 1.0).

## Discussion

This study provides a comprehensive evaluation of the performance of various LLMs on multiple domains, including readability, accuracy, comprehensiveness, response length, and response generation time, compared to retinal specialists in addressing real, unedited queries regarding retinal diseases. The findings underscore the potential and current limitations of AI-driven chatbots in the medical domain.

One of the primary observations was the notable difference in readability among the groups. Although responses generated by experts and Gemini exhibited higher FRES and lower FKGL, suggesting that these answers are more accessible to individuals with lower reading levels, they remain above the Brazilian average, which, according to the 2018 census, was 9.3 years of schooling [19]. Responses from ChatGPT 4.0, ConsensusGPT, and Claude 3, while demonstrating high accuracy and comprehensiveness, required even higher reading levels for comprehension. Our finding aligns with other published works that show readability indices with a relatively high difficulty level for chatbots, such as ChatGPT 4.0, and slightly easier readability in the case of Bard (currently Gemini) [20].

This discrepancy reflects not only an apparent trade-off—AI responses tend to be more detailed and of higher quality yet less accessible to lay audiences—but also shows that merely stating in the prompt that answers would be directed to ‘followers’ was insufficient to achieve a readability index appropriate for the general population. More explicit instructions to enhance readability might have produced different results, as previous studies demonstrated that requesting text with easier readability yields improvements in readability, especially for ChatGPT 4.0 [21]. Nonetheless it might be helpful to adjust the level of complexity of the response to the level of the question to avoid a discrepancy between the patient’s education level and the response, which has not yet been tested.

Regarding the answer quality and comprehensiveness, the LLMs-driven chatbots, particularly ChatGPT 4.0 and ConsensusGPT, achieved superior ratings compared to human experts and Gemini. Importantly, these performance patterns were consistent across both factual and judgment-based questions. In addition to accurate responses, ChatGPT 4.0 and ConsensusGPT provided additional context that contributed to a more comprehensive understanding of the queried topics. This finding is also echoed in current literature, where the quality of AI chat responses is comparable to or even superior to human responses, with ChatGPT 4.0 outperforming Gemini on more challenging issues related to retinal detachment [4, 11, 21]. The moderate but significant correlations between evaluators (τ = 0.317–0.337, *p* < 0.001) indicate reliable assessment of response quality, despite the inherently subjective nature of clinical judgment evaluation.

Response length and generation time further highlight the strengths and weaknesses of these systems. While AI-driven responses were generally longer, potentially contributing to their enhanced comprehensiveness, they were generated significantly faster than those provided by human experts. For instance, median response times for LLMs-driven chatbots ranged from 7 to 27 s, compared to 67 s for experts. This rapid response capability is a considerable advantage in clinical settings where timely information is paramount. However, the increased word count of some models (e.g., ConsensusGPT) may also necessitate balancing detail with brevity. It is important to note that the significantly shorter answers generated by Gemini were due to the manual configuration of this chatbot to provide short responses; had this change not been made, its responses would likely have been longer than those of other chatbots. The higher number of words in AI-generated responses compared to human responses is consistent with findings from other studies [6, 11]. Aditionally, regarding response length, our findings demonstrate that longer responses tend to receive higher quality and comprehensiveness ratings. While this relationship may reflect the capacity of longer responses to provide more comprehensive explanations and detailed information, it also raises concerns about potential evaluation bias, whereby assessors may unconsciously associate response length with quality. Furthermore, the absence of significant correlations in the LLM groups may be attributed to the limited variability of quality and comprehensiveness scores within those groups, which likely reduced the statistical power of the correlation analysis.

An additional aspect of the study was the ability of readers to distinguish between human-generated and AI-generated content. The high overall accuracy (83.13%) in identifying the source of the responses underscores that, despite the high quality of AI outputs, there remain discernible characteristics that differentiate them from human responses. In fact, the higher quality and completeness of the answers generated by artificial intelligence may have helped the evaluators identify the answers’ origin. This result is similar to another study that found an accuracy of 77.9% in identifying whether the author of the answers to ophthalmology-related questions was a specialist or ChatGPT 3.5 [22]. It is also interesting that Claude 3 and Gemini led to a higher number of misclassifications, suggesting that their responses may be more human-like than those generated by the other models, even so, when evaluating pairwise against the classification results for the Experts, the p-value was significantly low, demonstrating a statistically significant difference between the classification of these models compared to human responses.

Another noteworthy observation is that among the eighty evaluated answers generated by the AI systems, hallucinations—defined as instances where the model produces inaccurate or entirely fabricated information—were rare but not absent. One response introduced a fabricated element (Gemini’s response to question 19, which erroneously mentioned cyclopentane as a diagnostic gas), underscoring the need for caution when interpreting the apparent reliability of the generated content. Hallucinations have been documented in other studies, particularly when evaluating more extensive and diverse sets of queries [8, 16, 23]. Thus, while the frequency in our limited sample was low, the risk of hallucination may increase in broader applications or under different questioning conditions, underscoring the need for ongoing vigilance and quality control when integrating such technologies into medical practice.

The implications of these results are multifaceted. On the one hand, the high accuracy, comprehensiveness, and speed of AI responses indicate their potential as valuable tools in supporting healthcare professionals, particularly in settings where access to expert advice is limited. On the other hand, the elevated reading complexity of many AI-generated responses and the potential for hallucinations in larger datasets highlight the need for further optimization to ensure that the information is accurate but also accessible and reliable for a broader audience. Future work should explore strategies for simplifying AI outputs without compromising their quality and mechanisms for mitigating the risk of misinformation—especially when these systems are used directly by patients.

### Strengths and limitations

A major strength of the current study is that it focuses on a real-life, ongoing situation: patients interacting directly with chatbots in search of health information. In contrast, most earlier studies use expert-created questions to assess theoretical knowledge in an idealized manner and do not consider the communication difficulties that arise when patients seek information independently [5, 7, 20, 21, 24]. In addition, this is the most comprehensive yet to evaluate unedited answers from various artificial intelligence chats; the two published so far only assessed ChatGPT 3.0 [12] and ChatGPT 3.5 [11]. Furthermore, this is the first study to evaluate the readability and quality of chatbot responses to unedited questions on ophthalmology in Brazilian Portuguese, demonstrating that even in non-English contexts and complex scenarios involving direct patient interaction, these applications achieved superior results compared to human experts—a finding consistent with previous studies using expert-generated questions. It is worth noting that Brazil has one of the highest internet access rates worldwide [25].

Limitations of the current study include using a specific and limited set of retinal disease questions sourced from a single platform, which may not fully represent the diversity of inquiries encountered in everyday clinical practice. Moreover, while the study was conducted in Portuguese—a language with validated readability indices—the generalizability of the findings to other languages or cultural contexts warrants further investigation. The limited number of questions used to assess quality may have underestimated the likelihood of fabricated information, although at least one instance was identified in our sample. This remains a major concern when lay end-users access such applications. Finally, many other LLMs had emerged, including new versions of ChatGPT, DeepSeek, Grok and Claude 3.5, at the time of this manuscript’s submission, which warrants evaluation, as they may demonstrate even better performance.

## Conclusions

The current study demonstrates that LLMs-driven chatbots show great promise in generating accurate, comprehensive, and rapid responses to medical queries, interpreting questions even when they are poorly phrased and contain spelling and grammatical errors. Echoing the findings of previous studies, we observed that LLMs-driven chatbots can produce responses that are as good as, or even better than, those provided by human specialists, whether in ophthalmology or other areas of medicine. However, challenges remain in tailoring these outputs to be readily understood by all patient populations and ensuring the reliability of the information provided. Balancing the sophistication of medical information with accessible language and managing the risk of hallucinations will be key to maximizing the clinical utility of these tools.

## Supplementary Information

Below is the link to the electronic supplementary material.

Supplementary Material 1

## Data Availability

The datasets used and/or analyzed during the current study are available from the corresponding author upon reasonable request.
