# Supplementary material for: Chatbots versus retina specialists in answering real-world retina questions
Source: Int J Retina Vitreous. 2025 Oct 31;11:120. doi: 10.1186/s40942-025-00737-7 (PMC12577157; doi:10.1186/s40942-025-00737-7)
Supplement: Supplementary file 1 — Supplementary Material 1 [file 40942_2025_737_MOESM1_ESM.docx]

**Supplementary Table 1:** Twenty questions obtained from YouTube.

| **Question** | **Original Question in Portuguese** | **English Translation** | **Type** |
| --- | --- | --- | --- |
| 1 | Dr. Retirei o óleo de silicone, fez um mês, até agora minha visão não está boa. É normal? | Doctor, I removed the silicone oil a month ago, my vision is still not good. Is this normal? | J |
| 2 | Dr. boa tarde. Meu pai foi informado que tem sangramento nos olhos oque pode ser feito Dr.? Passaram para ele fazer laser, mas ele informa que sente dor quando Vai fazer esse laser e não continua o tratamento, fico triste demais com isso pq sei que a qualquer momento ele pode perder a visão, ele já é idoso 68 anos e insiste em andar de moto. Não sei oq fazer | Doctor, good afternoon. My father was informed that he has bleeding in his eyes, what can be done Doctor? They recommended laser treatment, but he says he feels pain when he goes to do this laser and doesn't continue the treatment, I'm very sad about this because I know he can lose his vision at any moment, he's already elderly 68 years old and insists on riding a motorcycle. I don't know what to do | J |
| 3 | Exame de fundo de olho e de retina sabe se tem essa doença (retinose pigmentar) ? | Can fundus and retinal examination detect this disease (retinitis pigmentosa)? | F |
| 4 | Ola doutor em agosto tive um deslocamento de retina e coloquei o oleo, agora em janeiro esta marcado pra retira, so que meu medico falou que seria bom coloca o gas assim que retira o óleo, isso me preocupa um pouco , E normal fazer tira o oleo e coloca gas | Hello doctor, in August I had a retinal detachment and had oil placed, now in January I'm scheduled to remove it, but my doctor said it would be good to place gas as soon as the oil is removed, this worries me a little, is it normal to remove the oil and place gas | J |
| 5 | Tenho retinose pegmentar, existe algum suplemento, medicamento ou procedimento para melhorar ou reverter minha visao? | I have retinitis pigmentosa, is there any supplement, medication or procedure to improve or reverse my vision? | F |
| 6 | Boa noite, Doutor, eu tive oclusão de ramo venoso com edema macular, isso significa que vou precisar tomar injeções anti-VEGF para sempre ou na maioria dos casos é possível ficar livre das injeções em definitivo? | Good evening, Doctor, I had branch retinal vein occlusion with macular edema, does this mean I will need to take anti-VEGF injections forever or in most cases is it possible to be free of injections permanently? | F |
| 7 | Serosa Central pode levar a cegueira? | Can Central Serous lead to blindness? | F |
| 8 | Boa Tarde Dr. Estou com 10% da visão, retirei o óleo de silicone faz 8 meses, será que a visão pode Melhorar ainda com mais tempo ainda? | Good afternoon Dr. I have 10% vision, I removed the silicone oil 8 months ago, can my vision still improve with more time? | J |
| 9 | Dr, apresento estes sintomas de metamorfopsia 2 meses e meio após a realização da vitrectomia por descolamento de retina. Tenho 42 anos. O que pode ser? | Doctor, I have these metamorphopsia symptoms 2 and a half months after vitrectomy for retinal detachment. I am 42 years old. What could it be? | J |
| 10 | Olá, boa noite, tenho uma pergunta pessoal: Eu tive oclusão de ramo venoso da retina com isquemia no olho direito e já tomei a minha primeira injeção de EYLIA há um mês, pela imagem de OCT aparentemente a mácula voltou ao normal apenas com UMA injeção e eu tive uma BOA melhora, mas minha visão ainda continua um pouco embaçada, o Senhor acredita que na próxima injeção eu posso melhorar ainda mais minha visão? | Hello, good evening, I have a personal question: I had branch retinal vein occlusion with ischemia in my right eye and I already took my first EYLEA injection a month ago, from the OCT image apparently the macula returned to normal with just ONE injection and I had a GOOD improvement, but my vision is still a little blurred, do you believe that with the next injection I can improve my vision even more? | J |
| 11 | Boa tarde. O estresse pode causar descolamento de retina? | Good afternoon. Can stress cause retinal detachment? | F |
| 12 | Olá doutor. Tenho sintomas de descolamento o que fazer até ir no oftalmologista? | Hello doctor. I have detachment symptoms, what should I do until I go to the ophthalmologist? | F |
| 13 | Oi tudo bem? Eu tenho 14 anos e umas semanas atrás eu mirei um laser no meu olhor direito por curiosidade e da queles laser de 2000w e agora eu estou com uma mancha borrada bem piquena no centro da minha visão direita oq eu faço agora? | Hi, how are you? I'm 14 years old and a few weeks ago I aimed a laser at my right eye out of curiosity and it was one of those 2000w lasers and now I have a very small blurred spot in the center of my right vision, what do I do now? | J |
| 14 | Existe pré disposição familiar à degeneração macular? Minha mãe e uma prima dela tem essa patologia | Is there a family predisposition to macular degeneration? My mother and a cousin of hers have this pathology | F |
| 15 | Tenho catarata e já tive descolamento de retina e o meu médico falou que é muito perigoso eu operar a catarata porque pode descolar a retina novamente eu gostaria de saber o que o senhor acha Minha retina descolou fazem 35 anos | I have cataracts and I've had retinal detachment and my doctor said it's very dangerous for me to operate on the cataract because it could detach the retina again, I would like to know what you think. My retina detached 35 years ago | J |
| 16 | Olá! Tive uma oclusão de veia central da retina em 04/07/23 e edema macular. Desde então, faço injeção intra vítrea de anti - vegf mensalmente. Após as 3 primeiras aplicações fiz um edema macular de repetição. Minha médica verificou na OCT que tenho uma membrana epirretiniana. Essa membrana pode atrapalhar o tratamento? É indicada cirurgia nesse caso? Cabe ressaltar que tenho glaucoma avançado em ambos os olhos e amaurose em OE | Hello! I had a central retinal vein occlusion on 04/07/23 and macular edema. Since then, I have monthly intravitreal anti-VEGF injections. After the first 3 applications I had recurrent macular edema. My doctor verified on OCT that I have an epiretinal membrane. Can this membrane interfere with treatment? Is surgery indicated in this case? It should be noted that I have advanced glaucoma in both eyes and amaurosis in the left eye | J |
| 17 | Tenho 16 graus de miopia e comeco de catarata se eu operasse resolveria meu problema? | I have 16 degrees of myopia and beginning cataracts, if I had surgery would it solve my problem? | J |
| 18 | Depois de aplicar avastin pode ser ter descolamento de retina !? | After applying Avastin can there be retinal detachment!? | F |
| 19 | Bom dia! Qual tipo de gás é usado no olho? | Good morning! What type of gas is used in the eye? | F |
| 20 | O que causa descolamento de retina? | What causes retinal detachment? | F |

Original question in Portuguese and its English translation. J indicates a judgment-based question, and F indicates a factual question.
